# Spatio-temporal Kriging for spatial irradiance estimation with short-term forecasting in a thermosolar power plant

**DOI:** 10.1016/j.heliyon.2024.e39247

**Published:** 2024-10-16

**Authors:** J.G. Martin, J.R.D. Frejo, J.M. Maestre, E.F. Camacho

**Affiliations:** a Department of Maritime and Transport Technology, TU Delft, Delft, The Netherlands; b Department of Systems Engineering and Automation, University of Seville, Seville, Spain

**Keywords:** Sensor networks, Kriging, Forecasting, Direct normal irradiance, Distributed estimation, Thermosolar plant

## Abstract

This article proposes a method to improve the efficiency of solar power plants by estimating and forecasting the spatial distribution of direct normal irradiance (DNI) using a sensor network and anemometer data. For this purpose, the proposed approach employs spatio-temporal kriging with an anisotropic spatio-temporal variogram that depends on wind speed to accurately estimate the distribution of DNI in real-time, making it useful for short-term forecast and nowcast of DNI. Finally, the method is validated using synthetic data from varying sky conditions, outperforming another state-of-the-art technique.

## Highlights

• An anisotropic spatio-temporal kriging approach is proposed to estimate/forecast DNI in a thermosolar plant on cloudy days.
• The method considers wind speed and direction, improving accuracy with fewer parameters than other estimation techniques.
• The proposed method outperforms state-of-the-art techniques in estimating DNI distribution and short-term forecasting.
• The approach is not only useful for thermosolar power plants but also has potential applications in photovoltaic plants.
• The technique can estimate/forecast other environmental variables, like pollutant concentration in flowing water.

## Nomenclature

**Acronyms**
**DNI**: Direct Normal Irradiance
**GIS**: Geographic information system
**CST**: Concentrated solar thermal plants
**HTF**: Heat transfer fluid
**TES**: Thermal energy storage
**MPC**: Model predictive control
**STV**: Spatio-temporal variogram

**Sets**
S: Set of sensors
V: Number of measurements
C: Set of cells
cc: Cell *c*
$s_{n}$: Sensor *n*
S: Number of sensors
M: Set of measurements
$m_{\mu }$: Measurement *μ*
$x_{\mu }$: *x* coordinate of *μ* measurement
$y_{\mu }$: *y* coordinate of *μ* measurement
$t_{\mu }$: *t* in which *μ* measurement was taken
$I_{\mu }$: DNI lecture of *μ* measurement

**Algorithm variables**
${\mathrm{CF}}_{ct}^{R}$: Real cloud factor in cell *ij* at time instant *t*
JtPS: Adjustment error of *PolyS* at time instant *t*
${\mathrm{CF}}_{ct}^{E}$: Estimated cloud factor in cell *ij* at time instant *t*
Et: Estimation error at time instant *t*
$\gamma^{D}$: Proposed STV
$\gamma^{\mathrm{PS}}$: *PolyS* STV
$\gamma^{M}$: Experimental STV
JD: Adjustment error of proposed method
JtD: Adjustment error of proposed method at time instant *t*
JPS: Adjustment error of *PolyS*

**Algorithm parameters**
RT: Range of instants in which a measurement affects other measurements
RD: Range of cells where a measurement affects other measurements
RM: Maximum number of measurements considered to estimate a certail cell DNI value
$h_{x,y,t}$: Spatial and temporal lags of measurements
$a_{1},a_{2},\ldots$: Parameters to adjust $\gamma^{D}$ to $\gamma^{M}$

**Other parameters**
N: Number of cells in the *X*-axis
M: Number of cells in the *Y*-axis

## 1 Introduction

Climate change is considered one of the top challenges for the future of humanity. For this reason, many society efforts aim at decreasing their levelized costs of electricity (LCOE) from renewable energies to beat those of fossil fuel technologies [1]. Therefore, it is not surprising that the use of solar energy around the world has rapidly increased in recent years. The most common plants for electric production using solar energy are photovoltaic plants (PV) and concentrated solar thermal plants (CST). In this regard, the LCOE of PV plants is substantially lower than that of CST plants, but CST plants can incorporate thermal energy storage (TES) (e.g., using molten salt) to generate electricity even during the night.

CST plants are usually located on large extensions of land with high solar incidence, with parabolic trough collector CST being the most cost-effective and common type [2]. These plants use parabolic mirrors that concentrate solar rays onto pipes located at the focal point of the parabola, where a heat transfer fluid (HTF) circulates and absorbs the concentrated solar radiation. The HTF is then sent to a power generation plant through a collector that gathers the hot oil from the manifolds. In most commercial plants, the solar field consists of a number of loops connected in parallel, with each loop formed by a number of serially connected collectors, usually four, which track the sun on one axis to maximize the collected energy [3]. Since the total flow rate through the collectors needs to be controlled to maintain the HTF temperature within operational limits while maximizing the power generated, a major challenge occurs when irradiance varies across the plant. For example, a localized cloud could trigger a harmful flow decrease in unshaded collectors, so their HTF temperature might go beyond the admissible levels, damaging the plant equipment. Because of this, in some cases it is necessary to defocus the collectors as a safety measure.

The problem of controlling CST flows has been addressed from different approaches, among which the best performance is achieved by the use of predictive control strategies such as model predictive control (MPC) [4], [5], [6], particularly when it is possible to control the flow entering each loop of collectors by means of valves. For these controllers, an estimation of the current and future distribution of the Direct Normal Irradiance (DNI) throughout the plant is needed. To this end, some authors such as [7] and [8] have proposed to use all-sky cameras to estimate the spatially distributed DNI from the images, and other authors such as [9], [10], and [11] have proposed the use of robot fleets to estimate it from the DNI measurements gathered.

In this context, the mapping of environmental variables has been largely approached using kriging [12], which has become a *de facto* standard for many geographic information systems (GIS) such as ArcGIS. Kriging is a technique that was first developed in [13] and has since become widespread for all types of spatial applications [14]. In particular, the spatio-temporal generalization is specially indicated for dynamic variables with shifting concentration over a certain area, becoming a suitable method to deal with DNI changes due to clouds. This technique also introduces significant advances in accuracy and applicability. Notably, existing approaches often rely on static spatial models that fail to capture the dynamic nature of environmental factors influencing solar irradiance. In contrast, these methods leverage both spatial and temporal data to account for rapid environmental changes. Indeed, this method has been proposed for simultaneous environmental mapping of dynamic variables in works such as [15], [16] and [17], and sensor placement in works such as [18], and [19]. [20], [21], and [22] proposed time-forward Kriging and vector autoregressive models to perform DNI forecasting. Some works also consider the effect of wind on the spatial distribution of DNI, e.g., [23] which highlighted the relevance of taking into account its direction and speed in this context, especially when carrying out short-term forecasting. However, this last method requires symmetry in the variogram, which is unreasonable with clouds moving in the wind direction, and employs a polynomial function to model the wind influence, losing accuracy in the estimation. Moreover, it also requires the estimation of a high number of parameters, particularly when many past measurements are used for the DNI prediction.

To deal with the above issues, this work proposes an innovative anisotropic spatio-temporal variogram (STV) that explicitly incorporates wind direction and speed into the model. These meteorological variables, readily measurable and routinely recorded by meteorological stations at most solar plant locations, improve the responsiveness of the STV to changing weather conditions. This integration enables more accurate and robust estimation and short-term forecasting, i.e., *nowcasting*, of the spatial distribution of solar irradiance. In addition, the proposed STV model achieves these improvements while requiring fewer parameters compared to traditional methods, which simplifies the computational process. Not only does this reduction in complexity make the model more efficient but it also increases its applicability in real-world scenarios, offering significant advantages in operational solar energy management. Additionally, a new method has been developed to select the measurements used in the kriging estimation, further refining the accuracy of our predictions. Finally, the versatility of the proposed method may also benefit PV plants, which can leverage distributed estimation and forecasting of DNI for enhanced operational efficiency.

The rest of this work is organized as follows: Section 2 details the formulation of the problem and describes how the proposed variogram was computed. Section 3 describes both how the data were generated and how the STV was adjusted to the data, and the results obtained for both real-time estimation and short-term forecasting. Finally, in Section 4 the conclusions of this work are listed and some future lines are discussed.

## 2 Spatial and temporal DNI mapping using Kriging

**Figure 1:**
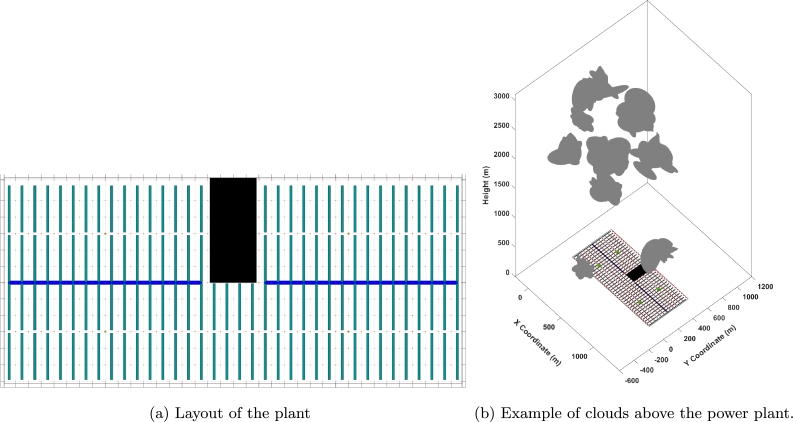
Thermosolar power plant scheme: (a) represents the layout of the plant and (b) represents the same layout in 3D under a certain distribution of clouds.

Kriging is a powerful geostatistical method that can be used for spatial and temporal estimation and prediction. It estimates the value of a variable at a specific location and time instant based on recent observations at nearby locations and time points [14]. Consider a PTC solar plant as the one shown in Fig. 1a, which is deployed over a very large flat area that can be discretized

into a meshgrid of N×M elements, yielding a set of *cells* $C=\{c_{1},c_{2}\ldots ,c_{c},\ldots ,c_{N\cdot M}\}$ whose DNI value at point $\left(x_{c},y_{c}\right)$ to be computed for each instant in a set $T\in \{t_{1},\ldots ,t_{T}\}$. It is assumed that there is a set of S DNI sensors $S=\{s_{1},s_{2},\ldots ,s_{S}\}$ located at different positions on the *XY* plane. Each sensor is assumed to continuously perform DNI measurements with metadata regarding their position and time, yielding a set of V measurements $M=\{m_{1},m_{2},\ldots ,m_{V}\}$, with $m_{\mu }=\{x_{\mu },y_{\mu },t_{\mu },I_{\mu }\}$ and μ=1,2,…,V. In this context, $x_{\mu }$ and $y_{\mu }$ denote the coordinates where the measurement *μ* was taken, $t_{\mu }$ indicates the time when the measurement was taken, and $I_{\mu }$ represents the DNI recorded.

Since the nominal DNI profile for a clean sky (no clouds or particles) can be computed using latitude, *solar day*, and *solar time*, as shown by [24], a *Cloud Factor* (CF) $CF_{ct}\in [0,1]$ is defined to measure the nominal DNI drop in the cell c∈C at time instant *t* when clouds are present, as in Fig. 1b. In particular, $CF_{ct}$ is 1 when a cloud eliminates 100% of the nominal DNI in a cell and 0 when the cell receives the nominal DNI. Intermediate values represent situations where the cloud is not dense enough to clog the nominal DNI completely.

The problem therefore is to compute an estimated/predicted CF in all cells c∈C and in all t∈T, say $CF_{ct}^{E}$, such that the total error:

$$E=\sum_{t\in T}E_{t},E_{t}=\frac{1}{N\cdot M}\sum_{c\in C}|CF_{ct}-CF_{ct}^{E}|,\;t\in T,$$

is minimized.

The estimation/prediction of the cloud factor at a location $\left(x_{c},y_{c}\right)$ and time instant *t* using kriging is performed as:

$$CF_{ct}^{E}=\sum_{\mu \in M^{\prime }(x_{c},y_{c},t)}w_{\mu }(x_{c},y_{c},t)\cdot I_{\mu },$$

where $w_{\mu }(x_{c},y_{c},t)$ are weights associated with each measurement in a measurement subset $M^{\prime }(x_{c},y_{c},t)=\{m_{1}^{\prime },m_{2}^{\prime },\ldots ,m_{V^{\prime }}^{\prime }\}\subset M$.^1^ These weights are computed solving the linear equations of ordinary kriging:

$$\left\{ \begin{array}{cc} \sum_{j\in M^{\prime }(x_{c},y_{c},t)}w_{i}(x_{c},y_{c},t)\cdot \gamma (x_{i}-x_{j},y_{i}-y_{j},t_{i}-t_{j})+u=\gamma (x_{i}-x,y_{i}-y,t_{i}-t),\; & i\in M^{\prime }(x_{c},y_{c},t)\\ \sum_{i\in M^{\prime }(x_{c},y_{c},t)}w_{i}(x_{c},y_{c},t)=1\; \end{array},\right.$$

where $x_{i}$, $y_{i}$, $t_{i}$ and $x_{j}$, $y_{j}$, $t_{j}$ are the locations and time instants of the *i*-th and *j*-th measurements, *u* is an additional variable to remove bias, and $\gamma (h_{x},h_{y},h_{t})$ is the spatio-temporal variogram (STV), which sets the semivariance for the lags in distance ($h_{x}$, $h_{y}$) and time ($h_{t}$). Here, $\gamma (h_{x},h_{y},h_{t})$ has been designed as (1):

(1) $$\gamma^{D}(h_{x},h_{y},h_{t})=\gamma_{0}^{D}+\gamma_{1}^{D}(h_{t})+\gamma_{21}^{D}(h_{t})\cdot \gamma_{3}^{D}(h_{x},h_{y},h_{t}),$$

where D stands for *designed*. In particular, $\gamma_{0}^{D}$ is the *nugget* effect (in this context, the standard deviation of the sensor), and $\gamma_{1}^{D}(h_{t})$, $\gamma_{2}^{D}(h_{t})$, and $\gamma_{3}^{D}(h_{x},h_{y},h_{t})$ are functions that depend on a set of parameters $a_{1},\ldots ,a_{10}$ and $b_{1}$ that must be adjusted:

• $\gamma_{1}^{D}(h_{t})$ models the purely temporal evolution of STV following a sigmoid that goes from $a_{1}$ to $a_{2}$ as $\left|h_{t}\right|$ grows, with $a_{3}$ and $a_{4}$ setting respectively its medium time and slope. Its expression is given by:
  $$\gamma_{1}^{D}(h_{t})=a_{1}+\frac{a_{2}}{1+e^{\frac{-(h_{t}+a_{3})}{a_{4}}}},$$
• $\gamma_{2}^{D}(h_{t})$ modulates the amplitude of $\gamma_{3}^{D}(h_{x},h_{y},h_{t})$ with the time lag $h_{t}$, and follows an inverse sigmoid that decreases to 0 as $h_{t}$ grows. Parameters $a_{5}$ and $a_{6}$ play an analogous role to that of $a_{3}$ and $a_{4}$. Its expression has been designed as:
  $$\gamma_{2}^{D}(h_{t})=\frac{b_{1}}{1+e^{\frac{h_{t}-a_{5}}{a_{6}}}}.$$
  Regarding its initial value, parameter $b_{1}$ is set as:
  $$b_{1}=-\gamma_{0}^{D}+\left(a_{1}+\frac{a_{2}}{1+e^{-\frac{a_{3}}{a_{4}}}}\right)\cdot \left(1+e^{-\frac{a_{5}}{a_{6}}}\right),$$
  to guarantee that $\gamma^{D}(0,0,0)=\gamma_{0}^{D}$, so that the variance of a measurement in the same spot and at the same time coincides with the *nugget effect* (the only uncertainty is that of the sensor). By setting $b_{1}$ in this manner, we ensure that the model's variance at the origin accurately reflects only the sensor's uncertainty, thus anchoring the model's response surface to a known baseline.
• $\gamma_{3}^{D}(h_{x},h_{y},h_{t})$ corresponds to a bidimensional Gaussian function given by:
  $$\gamma_{3}^{D}(h_{x},h_{y},h_{t})=\exp(-(h_{x}^{\prime }(h_{x},h_{t})+h_{y}^{\prime }(h_{y},h_{t}))).$$
  This function goes from 1 to 0 as the sum of the modified distance lags:
  $$h_{x}^{\prime }(h_{x},h_{t})=\frac{h_{x}-a_{7}\cdot v_{Wx}\cdot h_{t}}{a_{8}},\;h_{y}^{\prime }(h_{y},h_{t})=\frac{h_{y}-a_{9}\cdot v_{Wy}\cdot h_{t}}{a_{10}},$$
  grows. These lags set the center of the Gaussian function considering the displacement of the cloud in time, which is given by the product of $h_{t}$ and the wind speed components in the *X* and *Y* axis, $v_{Wx}$ and $v_{Wy}$, which are weighed by $a_{7}$ and $a_{9}$, and the distance lags $h_{x}$ and $h_{y}$, which are again weighed by parameters $a_{8}$ and $a_{10}$. The rationale of including the wind velocity is that the STV can be adjusted with data of any wind direction and velocity. Also, note that if there is total certainty in the wind direction and speed, $a_{7}$ and $a_{9}$ must be identical.

**Figure 2:**
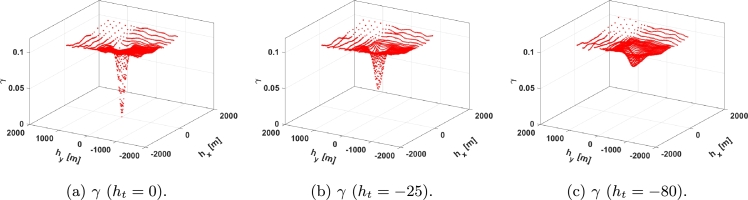
Temporal evolution of a STV: (a) represents the STV with the temporal lag being 0 seconds, (b) represents the STV with the temporal lag being 25 seconds, and (c) represents the STV with the temporal lag being 80 seconds.

Fig. 2 shows a STV. As can be seen, it becomes an upward-moving plane with a moving *bump* located at the spatio-temporal region where a measurement is more meaningful for the point and time where the cloud factor is to be estimated/predicted. That is, the lower the variogram, the stronger the effect of the corresponding measurements in the estimation/forecast. In general, *γ* increases with $\left|h_{t}\right|$ until it saturates, with the *bump* moving upwind pointing out the most relevant measurements. For example, if cloud shadows are moving eastward at 10 meters per minute and the cloud factor of point (x,y,t) is to be estimated using measurements only from 10 minutes ago, the most meaningful measurements will be those located 100 meters west of (x,y). However, as $\left|h_{t}\right|$ increases so does the minimum *γ*, since there is more uncertainty.

### 2.1 Identification of the STV parameters

Tuning the parameters of the proposed STV ($a_{1},\ldots ,a_{10}$) can be done as follows:

1. Select $N_{D}$ randomly distributed points in space-time.
2. For each random point $r=(x_{r},y_{r},t_{r})$, compute its experimental semivariance $\gamma_{r}^{M}$ in its surroundings using the cloud factor $CF^{R}(x,y,t)$ and that of the points surrounding it $CF^{R}(x+h_{x},y+h_{y},t+h_{t})$ using (2):
  (2) $$\gamma_{r}^{M}(h_{x},h_{y},h_{t})=\frac{1}{2}\cdot {\left(CF^{R}(x_{r}+h_{x},y_{r}+h_{y},t_{r}+h_{t})-CF^{R}(x_{r},y_{r},t_{r})\right)}^{2},\;h_{x}\in H_{x},h_{y}\in H_{y},\;h_{t}\in H_{t},$$
  where $\gamma_{r}^{M}$ is the *measured* gamma for point *r*, and $H_{x},H_{y}$, and $H_{t}$ are sets of predefined distances and time lags. Note that $H_{t}$ will only have negative values since, obviously, future measurements cannot be used. $H_{x}$ and $H_{y}$ will have negative and positive values with higher resolution upwind.
3. Use these $N_{D}\cdot |H_{x}|\cdot |H_{y}|\cdot |H_{t}|$ points to compute the $\left|H_{x}|\cdot |H_{y}|\cdot |H_{t}\right|$ points of the experimental STV by averaging, where |⋅| denotes the cardinality of the corresponding set, by (3):
  (3) $$\gamma^{M}(h_{x},h_{y},h_{t})=\frac{1}{N_{D}}\cdot \sum_{r=1}^{N_{D}}\gamma_{r}^{M}(h_{x},h_{y},h_{t}),\;h_{x}\in H_{x},h_{y}\in H_{y},h_{t}\in H_{t}.$$
4. Finally, the values of the parameters of the STV are obtained by minimizing the error between the designed STV $\gamma^{D}(h_{x},h_{y},h_{t}$) and the experimental one, $\gamma^{M}(h_{x},h_{y},h_{t})$, by (4):
  (4) $$J^{D}=\sum_{h_{t}\in H_{t}}J_{t}^{D},$$
  with
  $$J_{t}^{D}=\sum_{h_{x}\in H_{x}}\sum_{h_{y}\in H_{y}}|\gamma^{M}(h_{x},h_{y},h_{t})-\gamma^{D}(h_{x},h_{y},h_{t})|\;h_{t}\in H_{t}.$$
  Since this optimization is non-convex, it is necessary to run the optimization algorithm from many different initial points to avoid local minima.

### 2.2 The point of minimum semivariance and the selection of measurements

To estimate the cloud factor of point $\left(x_{c},y_{c}\right)$ at time *t*, it is necessary to define the *point of minimum semivariance* as the point with the most significant measurement according to the STV. Considering the STV proposed in (1), this point can be computed as:

$$p_{ms}(x,y,h_{t})=\left[\begin{array}{c} x_{ms}(x,y,h_{t})\\ y_{ms}(x,y,h_{t}) \end{array}\right]=\left[\begin{array}{c} x-\frac{a_{7}}{a_{8}}\cdot v_{Wx}\cdot h_{t}\\ y-\frac{a_{9}}{a_{10}}\cdot v_{Wy}\cdot h_{t} \end{array}\right]$$

**Figure 3:**
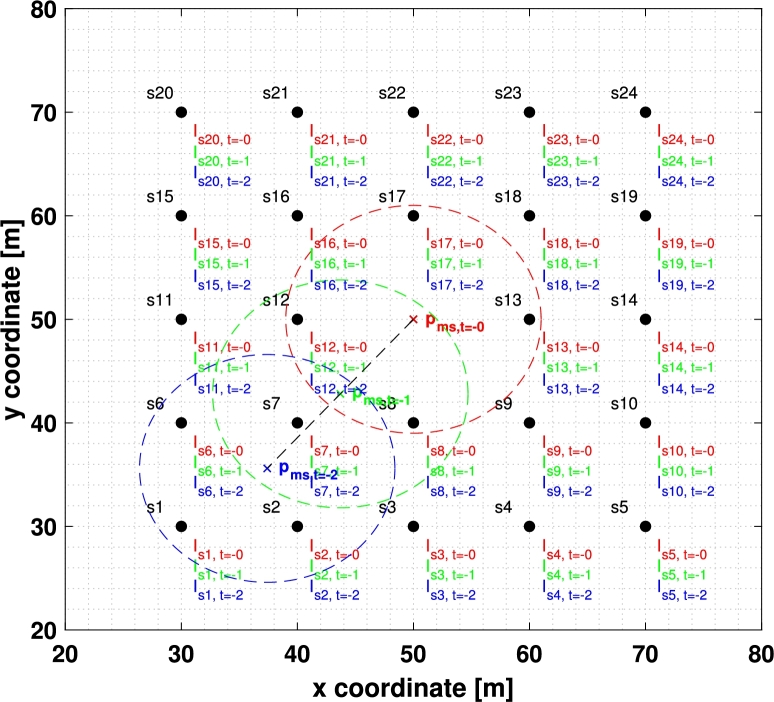
A mesh of 24 sensors takes 24 measurements per time instant with *R*_T_ = 2, i.e., only the current instant and the two previous instants are considered, having a total of V = 72 measurements (in red, green or blue depending on whether they were taken at *t*, *t* − 1 or *t* − 2 respectively). The position of the *point of minimum semivariance* in *t*, *t* − 1 and *t* − 2 is represented with a red, green and blue cross, and a circle with *R*_D_ radius is represented in the same color. Then, $M^{\prime }(x,y,t)$ will be composed of red measurements inside the red circle plus green measurements inside the green circle plus blue measurements inside the blue circle. Note that even though *s*_8_ and *s*_12_ are very close to the point where the estimation is wanted, it makes no sense to use measurements from these sensors of the instant *t* − 2.

Then, the measurement set $M^{\prime }(x,y,t)$ used for the estimation is selected as shown in Fig. 3. In particular, measurements are taken considering a temporal threshold, $R_{T}$, and a spatial threshold, $R_{D}$, based on their distance to $p_{ms}$. Finally, measurements are evaluated using $\gamma^{D}$ and sorted in decreasing order, so that the first $R_{M}$ are chosen.

## 3 Case study and results

In this section the case-study used to test the proposed method is presented. Particularly, Subsection 3.1 describes how the synthetic CF data were generated, Subsection 3.2 describes the process of adjusting the theoretic variogram in (1) to the data, in Subsection 3.3 the algorithm used for comparison is presented, and in Subsection 3.4 both algorithms are tested.

### 3.1 Data generation

Obtaining an accurate STV requires spatially distributed dynamic maps, which ideally involve a dense network of sensors. Such setups are cost-prohibitive due to high expenses in sensor deployment and maintenance. Therefore, generating data computationally provides a feasible and economical alternative that allows for extensive spatial analysis. Clouds are modeled as clusters of ellipsoids in random directions contained within a larger ellipsoid with random Gaussian dimensions following [25]; see, e.g., Fig. 1b. Wind is considered as a vectorial field in the volume contained within the limits of the plant area, the ground, and the maximum height of the cumulus clouds (2000 meters). It is important to keep in mind that the higher the altitude the faster the wind becomes, following the well-known Hellmann equation:

$$\frac{v_{W}}{v_{W}^{o}}={\left(\frac{h_{W}}{h_{W}^{o}}\right)}^{\alpha }$$

where $v_{W}$ is the speed of wind at height $h_{W}$ and $v_{W}^{o}$ is the speed of wind at the measurement height $h_{W}^{o}$; *α* is the Hellmann coefficient, which depends on the type of terrain. In this work, it is assumed that α=0.2, which corresponds to a moderately rough terrain.

Given the turbulent nature of the wind, it is also considered that in each of the mesh cells there is a disturbance $N(0,\sigma_{V})\in R^{3}$. Since the velocity field is available, the velocity of each cloud at each time instant can be interpolated. Then, Spencer equations [26] can be used to obtain solar rays from the center of each cell (here, cells are 20×20 $m^{2}$) and calculate their interference with clouds, assigning larger values of *CF* if the interference of the solar rays with a cloud is significant, and lower values otherwise. By generating random clouds and various wind fields as described above, different data sequences have been generated as shown here.^2^

### 3.2 Obtention of the variogram and adjustment to theoretical model

To compute the STV, $N_{D}=500000$ random points {xyt} have been generated and then a spatio-temporal mesh has been created surrounding each point, by defining $H_{x}$, $H_{y}$, $H_{t}$ (see (5)), to use (2) and (3).

(5) $$\begin{array}{cc} H_{x} & =H_{y}=[-1200,-1100,\ldots ,-100,-80,\ldots ,-20,0,200,\ldots ,1200]\;m,\\ H_{t} & =[-200,-180,\ldots ,-40,-30,-25,\ldots ,-10,-8,\ldots ,0]\;s. \end{array}$$

For the sake of accuracy, the mesh is denser upwind since the most significant measurements in the past are those in the upwind direction and farther away. In addition, as it was previously explained the significance of measurements decreases with time. Fig. 2 represents its spatial variation for $h_{t}=0$ seconds (Fig. 2a), $h_{t}=-25$ seconds (Fig. 2b), and $h_{t}=-80$ seconds (Fig. 2c) considering the velocity of the cloud shadows $v^{W}=[2,2]$.

**Figure 4:**
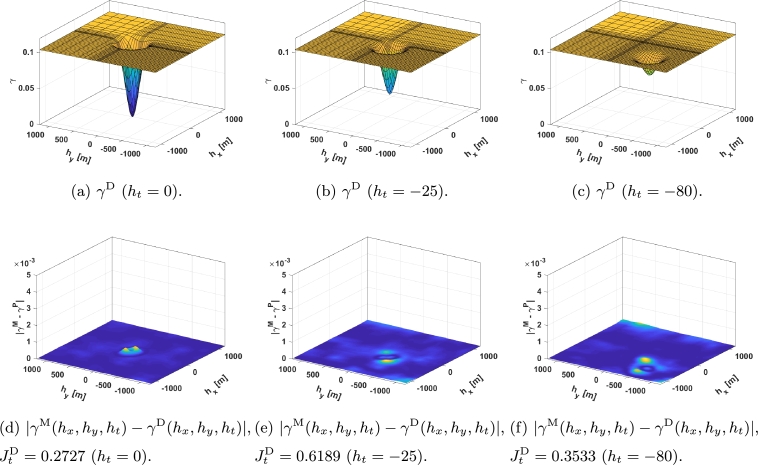
*γ*^D^ time slices: (a-c) represent the *γ*^D^ with the temporal lag being 0, 25, and 80 seconds respectively, and (d-f) represent the error with the STV with the same temporal lags.

Parameters $a_{1},\ldots ,a_{10}$ in equation (1) were adjusted using the fmincon optimization algorithm in MATLAB® to minimize equation (4), thereby obtaining the parameter values as listed in Table 1. The adjustment process consisted of employing SQP algorithms within fmincon to fine-tune the parameter values, in order to minimize the discrepancy between the model output and observed data, as defined by equation (1). For the sake of simplicity, $\gamma_{0}^{D}$ is set to 0 in this work. In Fig. 4, the adjusted proposed function, $\gamma^{D}(h_{x},h_{y},h_{t})$ (Fig. 4a-4c), along with $\left|\gamma^{M}(h_{x},h_{y},h_{t})-\gamma^{D}(h_{x},h_{y},h_{t})\right|$ (Fig. 4d-4f) are shown in the same temporal slices that were presented in Fig. 2.

**Table 1:** Adjusted parameters of the proposed spatio-temporal variogram.

*a*_1_	0.0994	*a*_6_	55.5886
*a*_2_	0.0047	*a*_7_	−0.2041
*a*_3_	40000.0921	*a*_8_	72.3831
*a*_4_	2001.8875	*a*_9_	−0.2041
*a*_5_	−589.0172	*a*_10_	80.2274

### 3.3 Baseline variogram and adjustment

**Figure 5:**
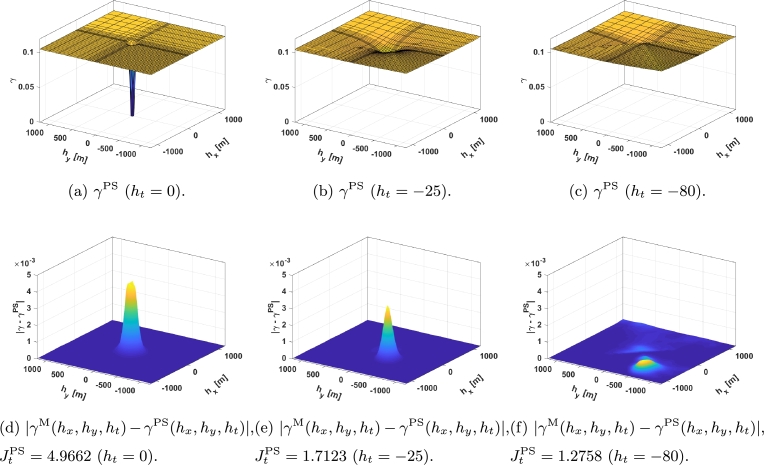
*γ*^PS^ time slices: (a-c) represent the *γ*^PS^ with the temporal lag being 0, 25, and 80 seconds respectively, and (d-f) represent the error with the STV with the same temporal lags.

The algorithm *PolyS* presented in [23] is used for comparison. This algorithm relies on the covariance function given by:

$$\begin{array}{cc} C_{\mathrm{PS}}(h_{1},h_{2},h_{t}) & =C_{\mathrm{FS}}(h_{1},h_{2},h_{t})+\lambda \cdot C_{\mathrm{Diff}}(h_{1},h_{2},h_{t}),\\ C_{\mathrm{FS}}(h_{1},h_{2},h_{t}) & =\frac{1-\nu }{1+a\cdot |h_{t}{|}^{2\cdot \alpha }}\cdot \left[e^{-\frac{c\cdot \sqrt{h_{1}^{2}+h_{1}^{2}}}{{\left(1+a\cdot |h_{t}{|}^{2\cdot \alpha }\right)}^{\beta /2}}}+\frac{\nu }{1-\nu }\cdot I_{h=0}\right],\\ C_{\mathrm{Diff}}(h_{1},h_{2},h_{t}) & =I_{|h_{t}|>0}\cdot I_{h_{1}>0}[K_{1}(|h_{t}|)\cdot h_{1}+K_{2}(|h_{t}|)\cdot |h_{2}|+K_{3}(|h_{t}|)\cdot h_{1}\cdot |h_{2}|+\\ & {+K_{4}(|h_{t}|)\cdot h_{1}^{2}+K_{5}(|h_{t}|)\cdot h_{2}^{2}+K_{6}(|h_{t}|)]}_{+}, \end{array}$$

with $h_{1}$ and $h_{2}$ the lag in the wind direction and its perpendicular respectively, I indicator functions, *ν* the nugget effect, and a set of parameters that need to be adjusted (*a*, *c*, *α*, *β*, *λ*, and a six *K*'s for each $h_{t}$ considered). Then, considering that the semivariogram and the covariance are related by $\gamma^{\mathrm{PS}}(h_{x},h_{y},h_{t})=C_{\mathrm{PS}}(\infty ,\infty ,\infty )-C_{\mathrm{PS}}(h_{x},h_{y},h_{t})$ and that the coordinates $h_{1}$, $h_{2}$, can be transformed into $h_{x}$, $h_{y}$ simply by a rotation, the same method as before can be used to adjust the parameters, i.e., fmincon in MATLAB® was used to minimize:

$$J^{\mathrm{PS}}=\sum_{h_{t}\in H_{t}}J_{t}^{\mathrm{PS}},$$

with

$$J_{t}^{\mathrm{PS}}=\sum_{h_{x}\in H_{x}}\sum_{h_{y}\in H_{y}}|\gamma^{M}(h_{x},h_{y},h_{t})-\gamma^{\mathrm{PS}}(h_{x},h_{y},h_{t})|\;h_{t}\in H_{t},$$

obtaining a=5.9254, c=1.2491, α=0.2786, β=0.4592, λ=4.6955, and, since the *nugget* effect is disregarded, $\nu =0=\gamma_{0}^{D}$. However, by using this method, one set of six *K*'s for each $h_{t}\in H_{t}$ is obtained. To obtain continuous *K* that allow us to use the STV with measurements at any temporal lag polynomial interpolation is used and the coefficients obtained can be seen in Table 2. In Fig. 5 the adjusted proposed function, $\gamma^{\mathrm{PS}}(h_{x},h_{y},h_{t})$ (Fig. 5a-5c), along with $\left|\gamma^{M}(h_{x},h_{y},h_{t})-\gamma^{\mathrm{PS}}(h_{x},h_{y},h_{t})\right|$ (Fig. 5d-5f) are shown in the same temporal slices that were presented in Fig. 2.

**Table 2:** *K* values in form *K*_*i*_ = *c*_1_ ⋅ |*h*_*t*_|^3^ + *c*_2_ ⋅ |*h*_*t*_|^2^ + *c*_3_ ⋅ |*h*_*t*_| + *c*_4_.

	*c*_1_ ⋅ 10^8^	*c*_2_ ⋅ 10^6^	*c*_3_ ⋅ 10^4^	*c*_4_ ⋅ 10^4^
*K*_1_	0.0114	−0.0393	0.0361	−0.3667
*K*_2_	0.0109	0.0382	0.0357	−0.05541
*K*_3_	−0.0002	0.0006	−0.0005	0.0035
*K*_4_	0.0001	0.0002	−0.0002	0.0023
*K*_5_	−0.0001	0.0002	−0.0002	−0.0004
*K*_6_	0.4505	1.5310	−1.4394	9.8246

A video containing $h_{t}\in H_{t}$ can be seen here.^3^ The graph shows the experimental values on the left, the proposed adjusted function in the second place, the error between the former and the obtained data in the third place, the adjusted *PolyS* in the fourth place, and finally, the error between the *PolyS* and the experimental data is depicted on the right.

### 3.4 Map estimation results

In this subsection a field of 5000×2000 meters (based on a typical CST), was meshed into a 250×50 grid, i.e., in 20×20 meters cells.

#### 3.4.1 Sensor meshes

The spatio-temporal Kriging was tested in 2 different scenarios: one with sensors every 500 meters (coarse mesh scenario) and another one with sensors every 200 meters (fine mesh scenario). The temporal range, the spatial range and the maximum number of measurements were adjusted manually in both scenarios, and their values can be seen in Table 3.

**Table 3:** Ranges for kriging.

	Fine Mesh of sensors	Coarse mesh of sensors
*R*_T_ [s]	300	300
*R*_D_ [m]	220	560
*R*_M_ [measurements]	40	200

#### 3.4.2 Random instant analysis

**Figure 6:**
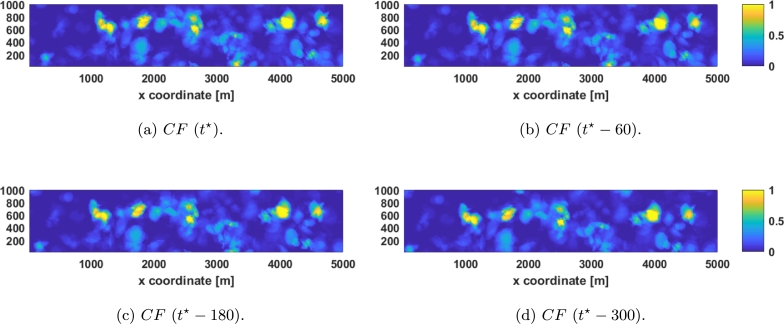
Simulated data in different interesting time instants. (a) represents the real CF in *t*^⋆^, (b) represents the real CF one minute before *t*^⋆^, (c) represents the real CF three minutes before *t*^⋆^, and (d) represents the real CF five minutes before *t*^⋆^. The video can be seen here.

In order to test the effectiveness of the algorithm a specific time instant $t^{⋆}=3670$ is randomly selected, which can be seen in Fig. 6a. Some previous instants are used to gain insight on the selected one, namely $t^{⋆}-60=3610$, $t^{⋆}-180=3490$, and $t^{⋆}-300=3370$, which are also shown in Fig. 6b, Fig. 6c, and Fig. 6d respectively. Note that instant 3370 is the least significant instant for their estimation since time range is 300 seconds. All these time frames can also be seen here.^4^

**Figure 7:**
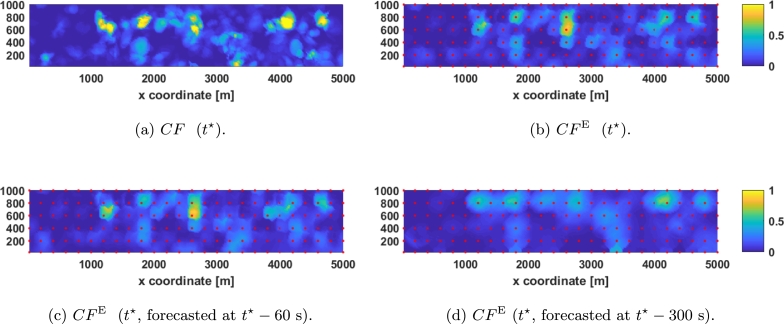
Comparison between kriging estimation with fine mesh and real DNI. (a) represents the real CF in *t*^⋆^, (b) represents the estimation obtained in *t*^⋆^, (c) represents the forecasting of *t*^⋆^ done one minute before, and (d) represents the forecasting of *t*^⋆^ done five minutes before. Red dots represent sensors of the fine mesh.

First, the ability of the fine mesh to estimate the complete map at $t^{⋆}$ (see Fig. 7a) has been tested (the results are shown in Fig. 7b) obtaining $E_{t}=0.05132$. Next, the very short-term (1 minutes in advance) forecast estimation is assessed obtaining $E_{t}=0.05591$, i.e., only 8.9% worse than the real time estimation (see Fig. 7c). Finally, for the short-term (5 minute in advance) forecast estimation (which can be seen in Fig. 7d) $E_{t}=0.08189$ is obtained, which is 58.56% worse than the current estimation. Note that the most relevant clouds (the ones around [1200,600], [2000,800], [2600,700], [4400,800], and [4600,800]) can be seen in the short term estimation.

**Figure 8:**
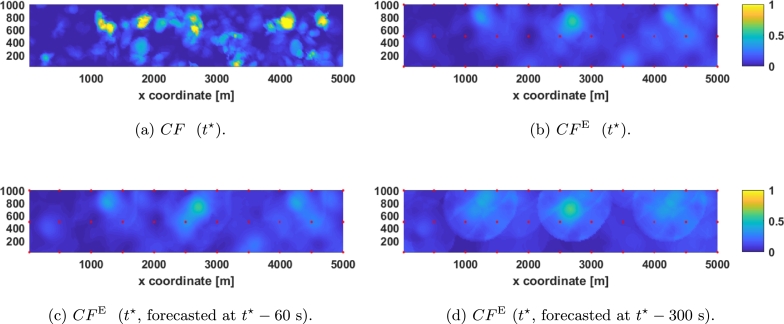
Comparison between kriging estimation with coarse mesh and real DNI. (a) represents the real CF in *t*^⋆^, (b) represents the estimation obtained in *t*^⋆^, (c) represents the forecasting of *t*^⋆^ done one minute before, and (d) represents the forecasting of *t*^⋆^ done five minutes before. Red dots represent sensors of the coarse mesh.

Then, the ability of the coarse mesh to estimate the instant $t^{⋆}$ (see Fig. 8a) is checked (results that can be seen in Fig. 8b) obtaining $E_{t}=0.08231$. Next, the very-short-term forecasting capability is checked by estimating the DNI along the plant 1 minute in advance (see Fig. 8c), obtaining an average error of $E_{t}=0.08794$, which is 6.85% worse than the real time estimation. Finally, the short-term forecasting capability (5 minutes in advance) using the coarse mesh is tested (see Fig. 8d) obtaining $E_{t}=0.09833$, which is 19.47% worse than the real time estimation.

As can be seen in Fig. 7 and Fig. 8, the estimation becomes fuzzier as the forecasting time increases. Also, note that the relative decrease in performance with this parameter in the coarse mesh is less than in the fine mesh, but the real time estimation is 60.37% worse with the coarse mesh.

#### 3.4.3 Averaging results

The $E_{t}$ in the two meshes was also compared with different forecasts, from 1 to 5 minutes during a 10-minute simulation from $t_{1}=337$ until $t_{T}=349$ (randomly selected) to study how it varies with it. The averaged results can be seen in Fig. 9 and a video can be seen here.^5^ Note that the error increases as the forecasting time does. However, this increase is greater with the coarse mesh than with the fine one.

**Figure 9:**
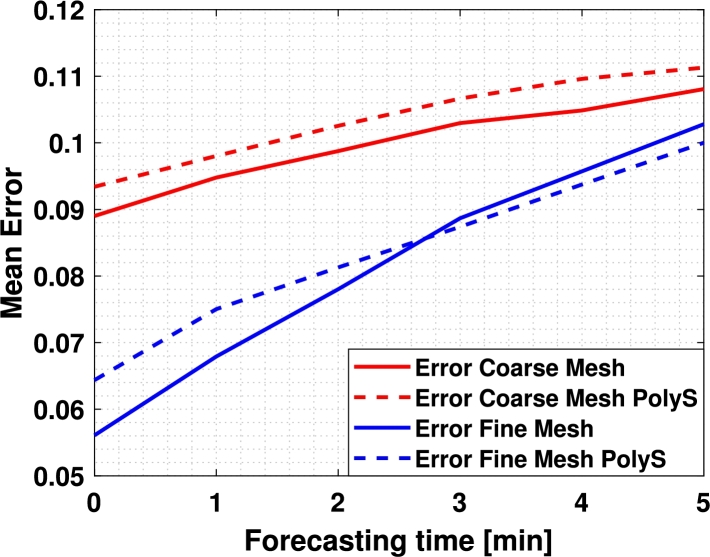
Mean error of the current estimation and the forecasted estimations with different forecasting times (1, 2, 3, 4, and 5 minutes) in both sensor meshes during the time interval that goes from *t*_1_ = 3370 seconds to *t*_T_ = 3970 seconds. A video of the current estimation and the forecasts during this time interval can be seen here.

**Figure 10:**
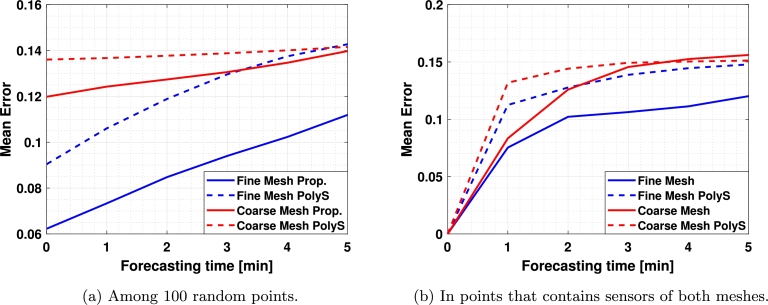
Average error during the complete simulation. (a) represents the average of the error among 100 random points, and (b) represents the average error on those points where there are sensors on both meshes.

Then, 100 random points were taken where the current estimation and 1, 2, 3, 4, and 5 minutes forecasts were performed during the 11 hours simulation and the error was averaged, obtaining the results in Fig. 10a. Also, in order to test the forecasting capacity of both algorithms in a point where there is a sensor, the same test was performed considering the 4 points where there is a sensor in both the fine and the coarse meshes ([1000,1000], [2000,100], [3000,1000], and [4000,1000]) obtaining the results in Fig. 10b.

Finally, a summary comparing both methods is shown in Table 4. It can be seen that the proposed method outperforms the one in the literature for the estimation and the forecasting except for the 5 minutes forecasting with the coarse mesh, where similar results were obtained. Also, it can be seen that the improvement is greater for estimation or *nowcasting*, rather than for short-term forecasting because performance decreases with forecasting time.

**Table 4:** Comparison of our proposal and the baseline method.

Prop./*PolyS*	Fine Mesh						Coarse Mesh					
Forecasting time [min]	0	1	2	3	4	5	0	1	2	3	4	5
Random points	0.69	0.69	0.71	0.73	0.74	0.78	0.88	0.91	0.92	0.94	0.96	0.99
In sensors	NA	0.67	0.80	0.77	0.77	0.81	NA	0.63	0.87	0.98	0.95	1.03

## 4 Conclusions

A novel anisotropic STV considering the direction and speed of the wind is presented here. The proposed method can be used to obtain spatially distributed estimation of DNI and short-term forecast estimations and outperforms another recent method in the literature for the considered application.

The spatio-temporal kriging technique provides an estimation of the variables measured and the standard deviation at each point of the map. Therefore, a similar strategy to the one in [11] can be carried out, paving the way to replace the wireless sensor network proposed here by a robotic sensor network. In addition, kriging also offers the possibility of integrating different types of data. Currently, other sources of data such as the temperature sensors of the loops are in the process of being included, which can help estimate the DNI received by the loops [27]. Furthermore, following [28] estimations of DNI through video cameras will be incorporated, which can be available at the plant at present and that can be integrated in aerial robots in the future. Finally, this method could be enhanced by machine-learning hybrid techniques as the ones presented in [29], [30], and [31].

## CRediT authorship contribution statement

**J.G. Martin:** Writing – review & editing, Writing – original draft, Software, Methodology, Investigation, Formal analysis, Data curation, Conceptualization. **J.R.D. Frejo:** Writing – review & editing, Writing – original draft, Validation, Supervision, Software, Data curation, Conceptualization. **J.M. Maestre:** Writing – review & editing, Writing – original draft, Visualization, Validation, Supervision, Resources, Methodology. **E.F. Camacho:** Validation, Supervision, Resources, Project administration, Methodology, Funding acquisition.

## Declaration of Competing Interest

The authors declare the following financial interests/personal relationships which may be considered as potential competing interests: Eduardo Fernandez Camacho reports was provided by 10.13039/501100000781European Research Council (grant agreement No 789051). Jose Maria Maestre Torreblanca reports financial support was provided by Spain Ministry of Science and Innovation (Grant no. PID2023-152876OB-I00). If there are other authors, they declare that they have no known competing financial interests or personal relationships that could have appeared to influence the work reported in this paper.

## Data Availability

All data required to support this study is available on request.

## Footnotes

1 For a given t∈T, forecasting is performed when time $t>\max_{\mu \in M^{\prime }(x,y,t)}t_{\mu }$; otherwise, estimation is performed.

2 https://youtu.be/LkV-yv38ONc.

3 https://youtu.be/ltqGvkWgvHc.

4 https://youtu.be/LkV-yv38ONc.

5 https://youtu.be/ss-3aXJRCuQ.
